# Supplementary material for: Disentangling the Ecological Processes Shaping the Latitudinal Pattern of Phytoplankton Communities in the Pacific Ocean
Source: mSystems. 2022 Jan 4;7(1):e01203-21. doi: 10.1128/msystems.01203-21 (PMC8725599; doi:10.1128/msystems.01203-21)
Supplement: TABLE S2 [file msystems.01203-21-st002.docx]

**Table S2. Sequence, OTU and alpha diversity information of samples.**

|  | **Size** | **Total seqs** | **Av. seqs** | **Total OTUs** | **Av. OTUs** | **Chao1** | **Shannon** | **Simpson** |
| --- | --- | --- | --- | --- | --- | --- | --- | --- |
| **Diatom** | 44 | 104988 | 2387 | 16327 | 785 ± 478 | 1467 ± 947 | 5.23 ± 0.82 | 0.96 ± 0.04 |
| **Syn** | 43 | 219292 | 5100 | 6343 | 320 ± 162 | 470 ± 247 | 2.39 ± 0.94 | 0.68 ± 0.21 |
| **Hapto.** | 45 | 108185 | 2404 | 6182 | 518 ± 306 | 1163 ± 907 | 4.33 ± 0.71 | 0.93 ± 0.04 |
