# Supplementary material for: Disentangling the Ecological Processes Shaping the Latitudinal Pattern of Phytoplankton Communities in the Pacific Ocean
Source: mSystems. 2022 Jan 4;7(1):e01203-21. doi: 10.1128/msystems.01203-21 (PMC8725599; doi:10.1128/msystems.01203-21)
Supplement: TABLE S3 [file msystems.01203-21-st003.docx]

**Table S3. Correlations between environmental/spatial factors and community dissimilarities.**

|  |  | **Env** | | **Geo** | | **Env\|Geo** | | **Geo\|Env** | |
| --- | --- | --- | --- | --- | --- | --- | --- | --- | --- |
|  |  | **Mantel-r** | ***p*** | **Mantel-r** | ***p*** | **Mantel-r** | ***p*** | **Mantel-r** | ***p*** |
| **Diatom** | **SUR** | **0.32** | **0.004** | **0.18** | **0.01** | **0.28** | **0.008** | 0.08 | 0.17 |
|  | **DCM** | **0.49** | **0.001** | **0.30** | **0.001** | **0.45** | **0.003** | **0.19** | **0.027** |
| ***Synechococcus*** | **SUR** | **0.59** | **0.001** | **0.44** | **0.001** | **0.52** | **0.001** | **0.31** | **0.001** |
|  | **DCM** | **0.51** | **0.001** | **0.34** | **0.001** | **0.54** | **0.001** | **0.40** | **0.001** |
| **Haptophytes** | **SUR** | **0.67** | **0.001** | **0.16** | **0.018** | **0.65** | **0.001** | 0.01 | 0.42 |
|  | **DCM** | **0.78** | **0.001** | **0.32** | **0.001** | **0.77** | **0.001** | 0.19 | 0.05 |
