# Supplementary material for: Disentangling the Ecological Processes Shaping the Latitudinal Pattern of Phytoplankton Communities in the Pacific Ocean
Source: mSystems. 2022 Jan 4;7(1):e01203-21. doi: 10.1128/msystems.01203-21 (PMC8725599; doi:10.1128/msystems.01203-21)
Supplement: TABLE S1 [file msystems.01203-21-st001.docx]

**Table S1. Location of sampling station and distribution of environmental factors.**

|  | **Lon, ^o^W** | **Lat, ^o^N** | **Tem** | **Sal** | **NO_3_** | **PO_4_** | **SiO_3_** | **NH_4_** | **PAR** | **MLD** | **ELD** | **Fe** |
| --- | --- | --- | --- | --- | --- | --- | --- | --- | --- | --- | --- | --- |
| **Surface layer** | |  |  |  |  |  |  |  |  |  |  |  |
| **NP1** | 170.05 | 0.28 | 28.25 | 35.4 | 5.09 | 0.6 | 1.43 | 0.28 | 47.62 | 51 | 70 | 0.16 |
| **NP2** | 169.92 | 4.97 | 29.33 | 34.23 | 0.04 | 0.21 | 0.31 | 0.07 | 30.87 | 74 | 94 | 0.46 |
| **NP3** | 170.12 | 10.03 | 28.46 | 34.77 | 0.02 | 0.25 | 0.35 | 0.08 | 41.19 | 76 | 103 | 0.14 |
| **NP4** | 170.02 | 15.01 | 27.5 | 34.99 | 0.02 | 0.2 | 0.37 | 0.06 | 53.14 | 91 | 116 | 0.12 |
| **NP5** | 169.92 | 20.02 | 27.49 | 35.16 | 0.06 | 0.18 | 0.05 | 0.05 | 53.14 | 40 | 100 | 0.28 |
| **NP6** | 170.02 | 24.08 | 26.49 | 35.57 | 0.01 | 0.08 | 0.24 | 0.05 | 56.39 | 27 | 113 | 0.15 |
| **NP7** | 170.02 | 30.00 | 25.95 | 35.23 | 0.02 | 0.05 | 1.47 | 0.06 | 59.23 | 21 | 93 | 0.24 |
| **NP8** | 170.02 | 35.03 | 23.77 | 34.59 | 0.02 | 0.07 | 2.5 | 0.07 | 55.93 | 13 | 70 | 0.12 |
| **NP9** | 170.00 | 40.03 | 16.7 | 33.71 | 0.46 | 0.22 | 2.39 | 0.22 | 48.21 | 24 | 63 | 0.12 |
| **NP10** | 170.07 | 45.05 | 12.82 | 33 | 12.6 | 1.33 | 20.74 | 0.21 | 40.23 | 15 | 64 | 0.09 |
| **NP11** | 170.03 | 50.00 | 11.59 | 32.56 | 10.3 | 1.29 | 26.34 | 1.11 | 34.65 | 19 | 63 | 0.08 |
| **NP12** | 170.00 | 55.00 | 11.36 | 32.6 | 1.43 | 0.84 | 3.52 | 1.1 | 34.83 | 15 | 31 | 0.12 |
| **NP13** | 170.07 | 60.00 | 9.81 | 30.44 | 0.07 | 0.44 | 2.68 | 0.46 | 36.71 | 16 | 29 | 2.74 |
| **NP14** | 168.00 | 64.25 | 9.11 | 31.16 | 0.15 | 0.35 | 0 | 2.21 | 32.3 | 12 | 20 | 0 |
| **NP15** | 168.00 | 68.00 | 4.05 | 32.32 | 1.09 | 0.81 | 0.14 | 0.21 | 34.73 | 15 | 27 | 7.11 |
| **SP1** | 170.00 | 0.00 | 27.3 | 35.49 | 5.59 | 0.56 | 2.56 | 0.34 | 52.12 | 15 | 53 | 0.23 |
| **SP3** | 170.00 | -10.00 | 30.42 | 35.13 | 3.77 | 0.53 | 1.81 | 0.06 | 38.56 | 47 | 102 | 0.18 |
| **SP4** | 170.07 | -15.00 | 29.06 | 35.26 | 0 | 0.21 | 1.23 | 0.07 | 47.29 | 27 | 96 | 0.36 |
| **SP5** | 170.03 | -20.02 | 27.75 | 35.43 | 0 | 0.14 | 1.15 | 0.08 | 53.9 | 47 | 94 | 0.29 |
| **SP6** | 170.02 | -25.00 | 26.28 | 35.55 | 0 | 0.06 | 0.98 | 0.08 | 51.48 | 25 | 98 | 0.19 |
| **SP7** | 170.00 | -30.00 | 24.01 | 35.44 | 0 | 0.05 | 0.99 | 0.06 | 46.53 | 15 | 115 | 0.06 |
| **SP8** | 170.00 | -35.00 | 21.66 | 35.28 | 0 | 0.08 | 1.04 | 0.04 | 53.55 | 31 | 121 | 0.24 |
| **SP9** | 170.00 | -40.00 | 19.78 | 35.07 | 0 | 0.15 | 1.13 | 0.08 | 48.81 | 32 | 101 | 0.36 |
|  |  |  |  |  |  |  |  |  |  |  |  |  |
| **DCM layer** | |  |  |  |  |  |  |  |  |  |  |  |
| **NP1** | 170.05 | 0.28 | 28.21 | 35.41 | 5.42 | 0.67 | 1.52 | 0 | 4.76 | 51 | 70 | 0.16 |
| **NP2** | 169.92 | 4.97 | 25.89 | 34.88 | 2.51 | 0.44 | 2.26 | 0 | 0.15 | 74 | 94 | 0.46 |
| **NP3** | 170.12 | 10.03 | 24.51 | 34.72 | 0.14 | 0.3 | 0.76 | 0.09 | 0.41 | 76 | 103 | 0.14 |
| **NP4** | 170.02 | 15.01 | 22.72 | 35.18 | 0.44 | 0.24 | 1.05 | 0 | 0.05 | 91 | 116 | 0.12 |
| **NP6** | 170 | 25 | 19.51 | 35.13 | 0.02 | 0.09 | 1.56 | 0 | 0.56 | 27 | 113 | 0.15 |
| **NP7** | 170 | 30 | 16.78 | 34.75 | 0.59 | 0.16 | 3.6 | 0.03 | 0.59 | 21 | 93 | 0.24 |
| **NP8** | 170 | 35 | 16.13 | 34.61 | 0.68 | 0.16 | 3.81 | 0 | 5.59 | 13 | 70 | 0.12 |
| **NP9** | 170 | 40 | 13.66 | 34.18 | 2.8 | 0.36 | 6.48 | 0.13 | 12.05 | 24 | 63 | 0.12 |
| **NP10** | 170 | 45 | 11.42 | 33.03 | 13.96 | 1.53 | 23.13 | 0.05 | 4.02 | 15 | 64 | 0.09 |
| **NP11** | 170 | 50 | 11.35 | 32.56 | 11 | 1.5 | 25.57 | 0.01 | 3.47 | 19 | 63 | 0.08 |
| **NP12** | 170 | 55 | 8.88 | 32.72 | 15.92 | 1.74 | 29.54 | 0.58 | 1.74 | 15 | 31 | 0.12 |
| **NP13** | 170 | 60 | 4.39 | 30.53 | 0.49 | 0.71 | 2.27 | 0.36 | 3.67 | 16 | 29 | 2.74 |
| **NP14** | 168 | 64.3 | 5.53 | 31.38 | 1.42 | 0.83 | 4.03 | 0.62 | 0.32 | 12 | 20 | 0 |
| **NP15** | 168 | 68 | 2.64 | 32.57 | 8.25 | 1.74 | 7.94 | 6.18 | 0.03 | 15 | 27 | 7.11 |
| **SP1** | 170 | 0 | 27.17 | 35.49 | 5.8 | 0.58 | 2.59 | 0.17 | 5.21 | 15 | 53 | 0.23 |
| **SP3** | 170 | -10 | 26.39 | 35.99 | 0 | 0.2 | 1.06 | 0 | 0.39 | 47 | 102 | 0.18 |
| **SP4** | 170 | -15 | 25.2 | 36.15 | 0.9 | 0.35 | 1.29 | 0.03 | 0.47 | 27 | 96 | 0.36 |
| **SP5** | 170 | -20 | 23.29 | 35.6 | 0 | 0.13 | 1.02 | 0 | 0.54 | 47 | 94 | 0.29 |
| **SP6** | 170 | -25 | 21.01 | 35.59 | 1.25 | 0.2 | 1.1 | 0.04 | 0.51 | 25 | 98 | 0.19 |
| **SP7** | 170 | -30 | 17.23 | 35.48 | 0.57 | 0.19 | 1.33 | 0.07 | 0.47 | 15 | 115 | 0.06 |
| **SP8** | 170 | -35 | 15.72 | 35.3 | 1.02 | 0.24 | 1.38 | 0 | 0.05 | 31 | 121 | 0.24 |
| **SP9** | 170 | -40 | 13.88 | 35.11 | 1.93 | 0.32 | 1.46 | 0 | 0.49 | 32 | 101 | 0.36 |
